# Responses of Terrestrial Ecosystems’ Net Primary Productivity to Future Regional Climate Change in China

**DOI:** 10.1371/journal.pone.0060849

**Published:** 2013-04-11

**Authors:** Dongsheng Zhao, Shaohong Wu, Yunhe Yin

**Affiliations:** Institute of Geographical Sciences and Natural Resources Research, Chinese Academy of Sciences, Anwai, Beijing, China; DOE Pacific Northwest National Laboratory, United States of America

## Abstract

The impact of regional climate change on net primary productivity (NPP) is an important aspect in the study of ecosystems’ response to global climate change. China’s ecosystems are very sensitive to climate change owing to the influence of the East Asian monsoon. The Lund–Potsdam–Jena Dynamic Global Vegetation Model for China (LPJ-CN), a global dynamical vegetation model developed for China’s terrestrial ecosystems, was applied in this study to simulate the NPP changes affected by future climate change. As the LPJ-CN model is based on natural vegetation, the simulation in this study did not consider the influence of anthropogenic activities. Results suggest that future climate change would have adverse effects on natural ecosystems, with NPP tending to decrease in eastern China, particularly in the temperate and warm temperate regions. NPP would increase in western China, with a concentration in the Tibetan Plateau and the northwest arid regions. The increasing trend in NPP in western China and the decreasing trend in eastern China would be further enhanced by the warming climate. The spatial distribution of NPP, which declines from the southeast coast to the northwest inland, would have minimal variation under scenarios of climate change.

## Introduction

The impact of climate change on ecosystems is an important topic that has elicited substantial interest across the world [1]. Analysis of recorded temperature in the past century shows a global surface average temperature rise of approximately 0.74°C. Based on the mean temperature recorded from 1980 to 1999, the global surface average temperature is projected to increase by about 1.1°C to 6.4°C by 2100 [2]. China’s climate, dominated by the East Asian monsoon, is extremely sensitive to global change [3]. Following projections from numerous general circulation models (GCMs), China would experience obvious climate changes in the future, including increase in average temperature, frequent occurrences of extreme climatic events, spatial and temporal heterogeneity in enhancing precipitation, and enlargement of its arid [4]. These changes in the climate can induce substantial variations in the composition, structure, and function of terrestrial ecosystems, thus inducing changes in ecosystem services, which are closely associated with the living environment of human beings and socioeconomic sustainability.

The impact of climate change on the ecosystem is poorly quantified because long-term in situ measurements are very sparse, and remote sensing techniques are only partially effective [2], [5]. Recent studies on the interaction between terrestrial ecosystems and climate change have primarily focused on enhancing the simulation of ecosystem models. Considering that ecosystem models can simulate not only the interaction among ecological processes but also the feedback between climate and ecosystems, these models are of paramount importance in understanding energy balance as well as the water and carbon cycles in an ecosystem [6]–[9]. Net primary productivity (NPP) is the rate at which carbohydrates accumulate in a plant’s tissues [10], [11]. NPP is not only an important index to describe an ecosystem’s structure and function but also a key element in describing carbon sequestration in an ecosystem during climate change [12].

Many models, including equilibrium biogeography and biogeochemical models, have been used in previous studies to simulate the impact of climate change on NPP in China. For example, Ni et al. employed an equilibrium biogeography model called BIOME3 to simulate changes in NPP under climate change scenarios [13]. The researchers found that NPP would increase in China from 2070 to 2099. Results from a process-based biogeochemical model (InTEC) indicated that the average forest NPP might be reduced from 2091 to 2100 under climate change, thereby inhibiting the CO_2_ fertilizing effect in plants [14]. Ji et al. found that the NPP of terrestrial ecosystems in China would decrease from 1991 to 2100 based on simulations by an atmospheric–vegetation interaction model (AVIM2) [15]. These discrepancies in NPP trends can be partially attributed to ecosystem models because most of them disregard the role of vegetation dynamics in the carbon cycle during climate change. Dynamic vegetation models, including vegetation dynamics and biogeochemical processes, have supplied us with an effective approach to project transient responses of the ecosystem to rapid climate change [16], [17]. Previous studies were almost entirely based on climate scenarios generated by GCMs with a resolution of 200 km to 300 km, which is too coarse for studies at regional or national scales. Regional climate models (RCMs) may be a better alternative in overcoming these shortcomings considering that these models provide greater spatial detail through dynamic downscaling of the GCM output [18].

Climate changes in China have significant regional differences; thus, the responses of terrestrial ecosystems to climate change vary in different regions. This study simulated the NPP of ecosystems in China under regional climate change scenarios (A2, B2, and A1B) based on a modified Lund–Potsdam–Jena Dynamic Global Vegetation Model (LPJ-DGVM). The temporal and spatial changes in the NPP of ecosystems in different regions were examined according to eco-regions. The purpose of the study was to elucidate the impacts of climate change on ecosystems at a regional scale and provide scientific basis for local adaptation and mitigating strategies.

## Methods and Data

### 1. Methods

LPJ-DGVM [19] is an integrated dynamic biogeography–biogeochemistry model developed based on an earlier equilibrium model, BIOME3. LPJ-DGVM is constructed in a modular framework, which combines process-based representations of terrestrial vegetation dynamics and land–atmosphere carbon and water exchanges. LPJ-DGVM explicitly considers key ecosystem processes such as vegetation growth, mortality, carbon allocation, and resource competition. Vegetation structure and composition are described by ten plant functional types (PFTs) (Table 1), which are distinguished according to their phenology, physiology, physiognomy, disturbance response attributes, and bioclimatic constraints. Gross primary production is computed based on a coupled photosynthesis–water balance scheme established through canopy conductance. Net primary production is calculated by subtracting autotrophic respiration. The sequestrated carbon is stored in seven PFT-associated pools representing leaves, sapwood, heartwood, fine roots, fast and slow decomposition in the aboveground litter pool, and a below-ground litter pool. The decomposition rates of soil and litter organic carbon depend on soil temperature and moisture. Model input includes monthly mean air temperature, total precipitation, number of wet days and percentage of full sunshine, annual CO_2_ concentration, and soil texture class. The full description of LPJ-DGVM was provided by Sitch et al. [19]; thus, only a short overview is provided in this paper. The LPJ-DGVM model is a typical dynamic vegetation model that has been widely utilized to study terrestrial ecosystem dynamics and interactions between climate change and ecosystems at global, regional, and site scales [8], [9], [16], [17], [20]. The version employed in the present study includes improved hydrology by Gerten et al. [20].

**Table 1:** PFTs Bioclimatic limiting factors used by LPJ-CN.

PFTs	*T_c_* min (°C)	*T_c_* max (°C)	GDD min (°C)	*T_w_* max (°C)
Tropical broad-leaved evergreen forest	*12.0* (15.5)			
Tropical broad-leaved raingreen forest	*12.0* (15.5)			
Temperate needle-leaved evergreen forest	−2.0	22.0	900	
Temperate broad-leaved evergreen forest	*0* (3.0)	*14.0* (18.8)	*1500* (1200)	
Temperate broad-leaved summergreen forest	−17.0	.*0* (15.5)	*1500* (1200)	
Boreal needle-leaved evergreen forest	−32.5	−*25.0* (2.0)	600	23.0
Boreal needle-leaved summergreen forest		−*2.0* (2.0)	350	23.0
Boreal broad-leaved summergreen forest		−*15.0* (2.0)	350	23.0
Shrub		−5.0	350	
Cold grass		−12.0		12
Temperate grass		*10.0* (15.5)		
Tropical grass	*15.0* (15.5)			

Note: The parameter values in round brackets given by Sitch et al. [19] are replaced by italic values in this study. Shrub and cold grass are newly added PFTs in LPJ. *T_c_* min the minimum coldest monthly mean temperature for survival; *T_c_* max the maximum coldest monthly mean temperature; GDD min the minimum growing degree days of over 5°C; *T_w_* max the upper limit of temperature of the warmest month.

In our previous study [21], LPJ-DGVM was carefully modified by adding shrub and cold grass PFTs, which were parameterized based on various inventory and observational data, with respect to the characteristics of ecosystems in China. The results of the simulations by the modified LPJ-DGVM for China (LPJ-CN) were validated with data sets obtained from the sites [21]. The simulated NPP results from LPJ-CN matched the observed data (R^2^ = 0.64, P<0.01), which was better compared with the original LPJ-DGVM data (R^2^ = 0.10) employed by Ni [22]. Therefore, LPJ-CN is assumed to be appropriate for simulating NPP in China.

Considering that the East Asian monsoon caused by differences in the heat-absorbing capacity of the continent and the ocean dominates the climate in China, climatic types vary from tropical in the south to cold temperate in the north and from humid in the east to dry in the west. Diverse climates and complex topography result in high biodiversity in China. The macro-spatial distribution of ecosystems forms different eco-regions with unique characteristics. Ecosystem responses to climate change in different eco-regions vary. According to the ecosystem regionalization scheme of China by Zheng et al. [23], China can be divided into eight eco-regions, namely, cold temperate humid region (I), temperate humid/sub-humid region (II), north semi-arid region (III), warm temperate humid/sub-humid region (IV), subtropical humid region (V), tropical humid region (VI), northwest arid region (VII), and Tibetan Plateau region (VIII). The impact of climate change on NPP was discussed in this paper according to the eco-region scheme.

According to the impact degree of future climate change on NPP, the change would be slight if the absolute change in NPP compared with the baseline term is <20%; the change would be moderate at 20% to 40%, the change would be severe at 40% to 60%, and the change would be extremely severe at over 60%. The slopes of linear regressions, obtained using the least squares method, were utilized to assess variation trends in the time series. Positive slopes indicate increasing trends, whereas negative slopes indicate decreasing trends. The significance levels of the trends were assessed by the non-parametric Mann-Kendall test.

### 2. Data

#### 2.1 Climatic data

The data on climate scenarios utilized in this study were provided by the climate change research group of the Institute of Environment and Sustainable Development in Agriculture, Chinese Academy of Agricultural Sciences. Based on the greenhouse gas emission scenarios of the IPCC Special Report on Emission Scenarios (SRES) [24], the group produced high-resolution (0.5°×0.5°) climate data scenarios for late 21^st^ century China through the Providing Regional Climate for Impacts Studies (PRECIS) system [25]. PRECIS was validated by applying reanalysis data derived from the European Centre for Medium-Range Weather Forecasts (ECMWF) as lateral boundary conditions; PRECIS was found to simulate terrestrial climate change in China effectively [26]. Projected climatic data, including three SRES emission scenarios (A2, B2, and A1B), for the period 1961 to 2100 were employed in the present study.

**Figure 1:**
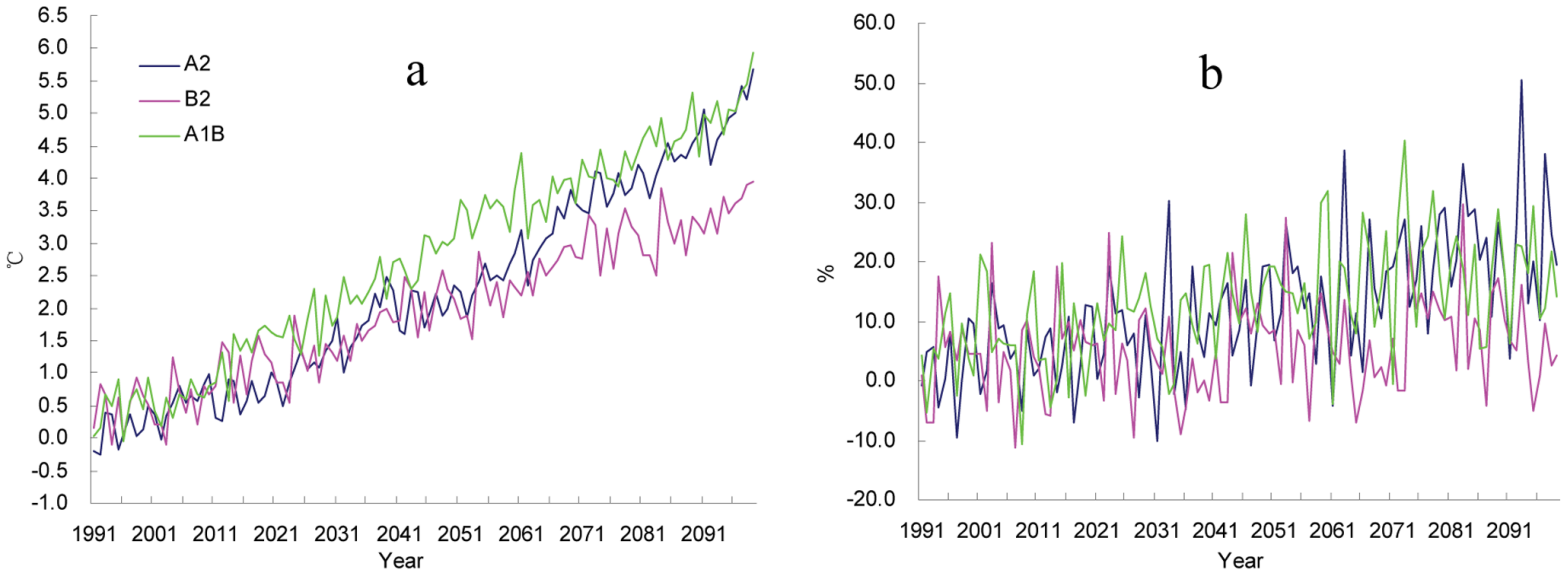
Inter-annual variations of temperature (a) and precipitation (b) anomalies averaged across China (relative to the reference averages from 1961 to 1990, projected by the PRECIS under SRES A2, B2, and A1B scenarios).

Compared with the baseline climate (1961–1990), the annual mean temperature of China (Fig. 1a) is projected to increase from 3.26°C (B2) and 4.64°C (A1B), with the highest increase in northwest and northeast China and the lowest increase in southeast China. The total annual precipitation across China (Fig. 1b) is projected to increase from 8% (B2) and 21% (A2), with the highest increase in northwestern China. A slight decrease in precipitation would occur in northeast China.

The periods were divided into baseline period (1961–1990), near term period (1991–2020), middle term period (2021–2050), and long term period (2051–2080). Each term was discussed according to the average of 30 years.

#### 2.2 Soil data

This research adopted soil texture data from the map of soil texture types (1∶14,000,000) [27], which contains information on the proportions of mineral grains in the top soil and geographical distribution of the different soil texture types. The soil textures were reclassified as clay, silt, sand, silty sand, sandy clay, silty clay, and clay with silt and sand according to the FAO classification standard for soil texture to address the data input requirements of LPJ-CN [28]. The soil data were then transformed into the ArcInfo grid format and resampled to the spatial resolution of 0.5°×0.5°.

### 3. Modeling Protocol

LPJ was operated for a “spin up” period of 900 years to achieve equilibrium in stable vegetation structures and C pools. The model was thereafter subjected to transient climate. Observed atmospheric CO_2_ concentration was applied to the ecosystem model simulation for the period of 1961 to 1990. In the projected simulation, the atmospheric CO_2_ concentration would remain at the value of 1990. The scheme was designed to eliminate the fertilizing effects of atmospheric CO_2_ concentration on the ecosystem.

## Results

### 1. Variation Trend of NPP in Different Eco-regions

**Figure 2:**
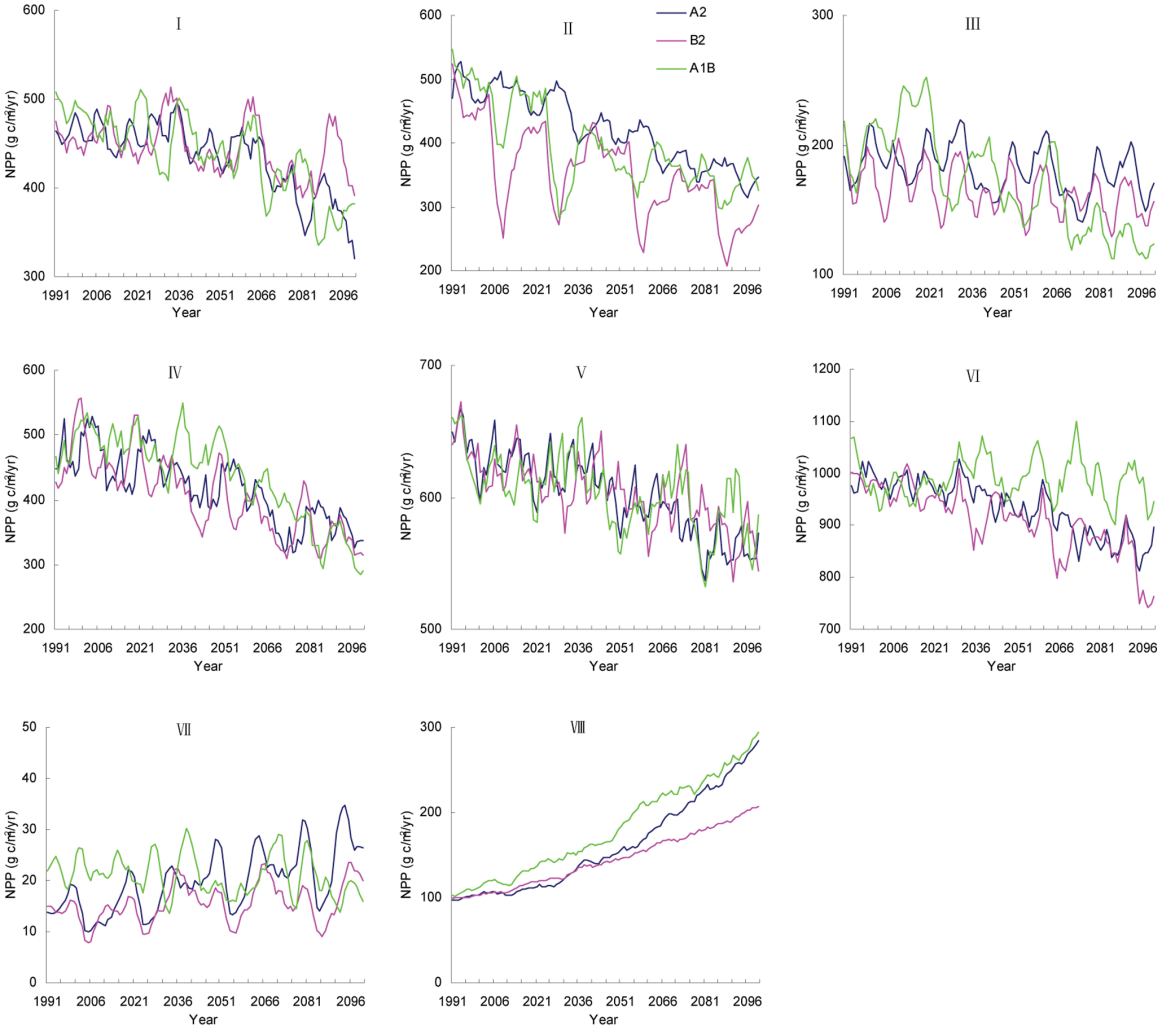
Inter-annual variation of NPP in different eco-regions (I refers to cold temperate humid region; II, temperate humid/sub-humid region; III, north semi-arid region; IV, warm temperate humid/sub-humid region; V, subtropical humid region; VI, tropical humid region; VII, northwest arid region; VIII, Tibetan Plateau region.) from 1991 to 2100 under SRES A2, B2 and A1B scenarios.

Figure 2 illustrates the inter-annual variations of average NPP in eight eco-regions under A2, B2, and A1B scenarios from 1991 to 2100. In the future, NPP may exhibit a significant decreasing trend in all eco-regions except in the Tibetan plateau and northwest arid regions. This projection is associated with the spatial heterogeneity of climate change and sensitivity of the ecosystem to climate change. In cold temperate humid region, the NPP trend can be distinguished in two periods: before and after 2030. From 1991 to 2030, NPP would remain unchanged but would significantly decrease after 2030 (Fig. 2). NPP in the temperate humid/sub-humid region is projected to decline at a rate greater than 1.5 g C m^−2^ yr^−1^ and would demonstrate a large decadal fluctuation particularly in the B2 and A1B scenarios. The largest decrease is projected to occur in the warm, temperate humid/sub-humid region then in the tropical humid and sub-tropical humid regions (Table 2). The various climate trends in the three climate scenarios can produce slight differences in the NPP trend for each eco-region. Considering that the average NPP in the three eco-regions is higher than that in the other eco-regions, the rapid decrease could have a significant contribution to the decline of NPP in entire China. Increase in NPP may occur in the Tibetan plateau and northwest arid regions due to warming climate. The largest increase in NPP may be found in the Tibetan plateau region, with an average NPP of 1.7 g C m^−2^ yr^−1^ for the A2 and A1B scenarios. The NPP increment in the northwest arid region may be slight; approximately 0.13 g C m^−2^ yr^−1^ (R^2^ = 0.46, P<0.001) for A2 and 0.14 g C m^−2^ yr^−1^ (R^2^ = 0.14, P<0.001) for B2. The annual variations in NPP are projected to exhibit a mild decreasing trend in the A1B scenario, although this projection did not pass the statistical tests.

**Table 2:** NPP Slopes (g C m^−2^ yr^−1^) in different eco-regions and its coefficient of determination under different climate scenarios.

Eco-regions	A2		B2		A1B	
	Slope	R^2^	Slope	R^2^	Slope	R^2^
I	−0.95	0.61	−0.37	0.17	−1.11	0.64
II	−1.63	0.87	−1.53	0.50	−1.59	0.60
III	−0.19	0.11	−0.24	0.18	−0.94	0.62
IV	−1.36	0.66	−1.49	0.64	−1.76	0.71
V	−0.83	0.74	−0.60	0.55	−0.53	0.36
VI	−1.43	0.75	−1.65	0.70	−0.05	0.01
VII	0.13	0.46	0.04	0.14	−0.03*	0.01
VIII	1.66	0.97	1.00	0.99	1.69	0.98

Note: *means the significance level is P<0.5, or else P<0.001.

### 2. Spatial Variation of NPP

**Figure 3:**
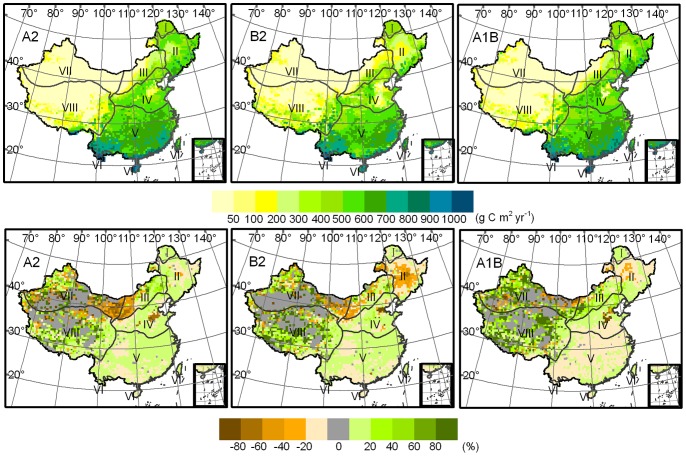
NPP spatial pattern (g C m^2^ yr^−1^) (upper panel) in the near term and its anomalies (%) (lower panel) with baseline over China modeled through climate change projections for SRES A2, B2, and A1B scenarios.

Figure 3 shows the spatial pattern of NPP in near term and its variation compared with the baseline term. The NPP in the majority of the ecosystems is projected to increase in near term in over 60% of the total land area of China under the A2, B2, and A1B scenarios. A slight increase in NPP is likely to be predominant. Over-moderate increment may exist only in a few ecosystems concentrated in the Tibetan Plateau and northwest arid region. A few ecosystems may experience a decrease in NPP particularly under the A1B scenario, which shows the largest area of NPP decrement amounting to approximately 37% of the overall territorial ecosystems. A slight decrement dominates the largest areas of ecosystems with decreasing NPP, accounting for 62%, 64%, and 84% in the A2, B2, and A1B scenarios, respectively. Only a few ecosystems are likely to have a rate of decrement over moderate levels; these ecosystems are mainly distributed in the north semi-arid and northwest arid regions, particularly in the A2 scenario.

**Figure 4:**
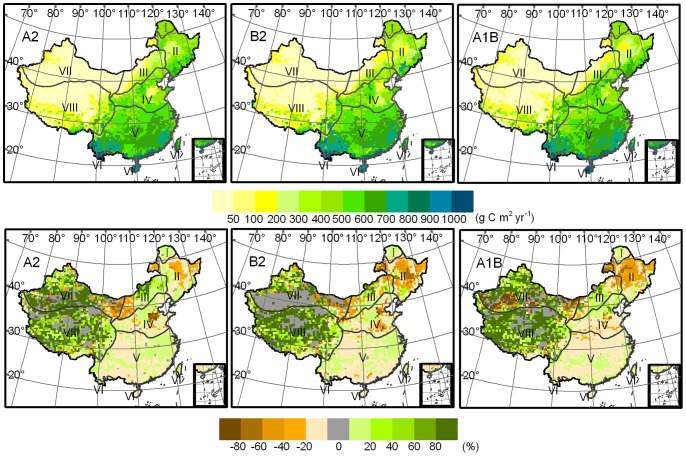
NPP pattern (g C m^2^ yr^−1^) (upper panel) in the midterm and its anomalies (%) (lower panel) with baseline over China modeled through climate change projections for SRES A2, B2, and A1B scenarios.

The trends of NPP, decreasing in east China and increasing in west China, are more obvious in the middle term compared with the near term (Fig. 4). A slight NPP decrement may dominate China’s ecosystems in the A2, B2, and A1B scenarios, occupying 25% and 26% of the overall territorial ecosystem in the A2 and B2 scenarios, respectively, which mainly occurs in the sub-tropical humid and warm temperate humid/sub-humid regions. In addition, ecosystems with over-moderate NPP decrement are limited to the temperate humid/sub-humid, north semi-arid, and northwest arid regions. Severe and extremely severe NPP decrements primarily exist in the central temperate humid/sub-humid region and the adjacent areas between the north semi-arid and northwest arid regions. The ecosystems with NPP increment are likely to decline, with approximately 53% of the total ecosystem in the B2 and A1B scenarios and 59% in the A2 scenario. In the B2 scenario, the area with a slight increment in NPP is reduced to 21% of the overall ecosystem, which is smaller than the value in the near term. Ecosystems with over-moderate NPP increment are still concentrated in the Tibetan Plateau and northwest arid region where the absolute NPP value is only 60 g C m^−2^, a value that is considerably lower than those in the other regions.

**Figure 5:**
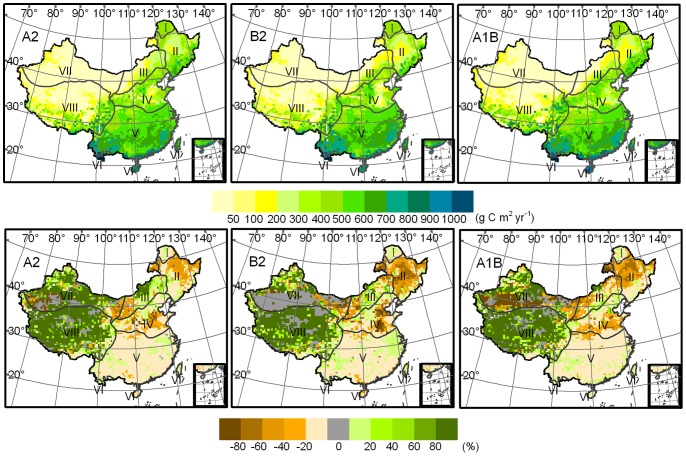
NPP pattern (g C m^2^ yr^−1^) (upper panel) in the long term and its anomalies (%) (lower panel) with baseline over China modeled through climate change projections for SRES A2, B2, and A1B scenarios.

Figure 5 illustrates the NPP distribution in the long term climate and change in NPP relative to the baseline term climate. In the A1B, A2, and B2 scenarios, the ecosystems with NPP decrement are expected to occupy 47%, 46%, and 52% of total area in China, respectively, which means that nearly half of the ecosystems in China may be exposed to the adverse effects of climate change. An NPP decrement may occur primarily in eastern China, which has an inherently high NPP. An NPP increment may occur in western China, which has low NPP. Ecosystems with slight NPP decrement would still predominate in China and would retain spatial patterns similar to that in the middle term. Areas with over-moderate NPP decrement are likely to expand further and are projected to become distributed in the temperate humid/sub-humid and warm temperate humid/sub-humid regions. Ecosystems may be severely impacted by climate change in the A1B scenario, and the area with the extremely severe NPP decrement would be greater than 7% of the overall territorial ecosystems. This projection is expected to occur in most parts of northern China. On the contrary, the NPP increment would be mainly concentrated in western China, particularly in the Tibetan Plateau region where the average NPP increment may reach approximately 60%. The NPP decrement in East China and increment in West China may be enhanced further in terms of spatial variation.

## Discussion

### 1. Effects of Climate Change on NPP

The projected climate change characterized by warm and wet climate may cause variations in NPP in most areas of China according to our simulated results. Regional differences are mainly associated with the combinations of variations in temperature and precipitation in all three scenarios. The NPP decrease in cold temperate humid regions may be associated with the rising temperature because a slight increase in temperature can benefit the boreal forest growth, whereas a significant increase in temperature may reduce the water effectiveness through thawing frozen soil and enhancing evapotranspiration, which in turn can result in negative effects for the boreal forest growth [15], [16], [29]. The large annual variation in NPP in the temperate humid/sub-humid region can be attributed to frequent drought derived from the climate scenarios, particularly in B2. The decrement in precipitation can impair the photosynthesis rate of vegetation in the model because of reduced stomatal conductance [30], [31].

The NPP decrement in warm temperate, sub-tropical, and tropical humid regions can be explained by increased water stress as a consequence of increased evapotranspiration that is not counterbalanced by an increase in rainfall. Piao et al. [29] also found that increase in temperature alone does not benefit vegetation NPP in temperate and tropical ecosystems because of water control. Studies on the historical tree ring records of tropical forests and temperature [32], [33] suggest that water availability is a key limiting factor that controls vegetation NPP. Given that water limits photosynthesis in plants in the northwest arid region where plants are more sensitive to precipitation variation than temperature variation, increasing precipitation can result in an increase in carboxylation efficiency, which is the primary cause of enhanced productivity [30]. The NPP in the A1B scenario exhibits a different trend from that in A2 and B2. This different trend could have been caused by different precipitation trends. Before 2030, the A1B scenario has a greater amount of rainfall than the other two scenarios; however, the amount of rainfall would remain unchanged or even decrease after 2031. The relatively low temperature in the Tibetan Plateau limits plant growth, but warming may solve this limitation, thus leading to a general increase in NPP [13], [34].

The results of this research are generally consistent with the results of previous studies [15], [14], [28], [35]. However, some discrepancies exist. Our study suggests that NPP in eastern China, which is covered with forests, is likely to decrease even with the influence of climate change. This is similar to the result obtained by Ju et al. [14]. The spatial change in NPP, namely, a decrease in eastern China and an increase in western China under the climate change scenarios, is consistent with Wu et al. [35] and Ji et al.’s [15] results. The NPP changes in several sensitive areas located at the temperate humid/sub-humid, warm temperate humid/sub-humid, and north semi-arid regions are different from the results of previous studies [15], [35]. These areas have an increasing incidence of extreme climatic events; therefore, biogeography and biogeochemical models cannot simulate the effects of extreme climatic events on ecosystems. Dynamic vegetation models can capture these effects, leading to discrepancies in results.

### 2. Uncertainties in the Study

LPJ-CN, a process-based ecosystem model, was applied in this study to simulate NPP changes in China under scenarios A2, B2, and A1B. The study examined the NPP variation in three projected periods, which improves the understanding of the impact of the different warming levels. However, several uncertainties underlying future climate change and ecosystem responses exist. The trends of climate change in China are different in various climatic scenarios. However, we only probed the possibilities for the IPCC SRES A2, B2, and A1B scenarios. Given that the climatic system is complicated and nonlinear and China’s landform is very complex with altitudes varying from −50 m to 8843 m, GCMs are often unable to depict the accurate spatial patterns of the climatic factors employed in ecosystem modeling with low resolution. Although the simulation capability of PRECIS with boundary condition from the general circulation model (GCMs)-HadAM3 [25] has been improved in this study by enhancing spatial resolution, the aforementioned problems still cannot be addressed entirely to eliminate uncertainties. Moreover, some uncertainties remain with regard to the parameterization and process representations in the LPJ-DGVM model [36]. For example, the model does not provide an explicit representation of the nitrogen cycle; some scientific assumptions are unreasonable, and some lagging responses and self-adaptive mechanisms of the ecosystem to climate change are omitted in the LPJ-DGVM model. These issues have attracted the attention of researchers around the world, but there are insufficient studies to support the quantification of these processes in the model.

The LPJ-CN model predicts the temporal and spatial change of ecosystem NPP according to potential natural vegetation, which is free from the influence of anthropogenic activities. However, the great expansion in human activity can definitely have a major impact on ecosystem dynamics [37]. Liu et al. reported that approximately 5.2 Mha of grassland and woodland in China were converted to cropland in the 1990s because of reclamation activities [38]. Piao et al. found that the large-scale reforestation and afforestation programs since the 1980s in China have resulted in the increase of forest biomass carbon stocks [39]. Considering the lack of information on the amount and spatial patterns of land use in the future, the accurate estimation of the magnitude of NPP change associated with land use remains a challenge [40]. These uncertainties can be addressed by integrating human disturbances in future studies.

### Conclusions

We utilized a dynamic vegetation model, LPJ-CN, to simulate the temporal and spatial responses of ecosystem NPP to climate change in 21st century China under A2, B2, and A1B scenarios. Our results indicate that rising temperature and slightly changing precipitation would lead to a serious impact on the natural ecosystem’s NPP in general. The near-term, mid-term, and long-term impact of climate change may become worse gradually. In the near term, the impact of climate change on the ecosystem may be minimal; more than half of the ecosystems may benefit from the change. However, an adverse impact may occur in the middle term. In the long term, more than half of ecosystems may be exposed to adverse effects. NPP enhancement may appear mainly in the Tibetan Plateau and northwest arid region. The other eco-regions may be dominated by NPP increase. Although NPP may increase by a relatively high percentage in the Tibetan Plateau and northwest arid region, it cannot generate a significant influence on the overall NPP distribution in China because of its low initial productivity level. Therefore, the spatial distribution of NPP, which decreases from the southeast coast to the northwest inland, would not be altered under the climate change scenarios. The response of China’s terrestrial ecosystems to climate change will contribute to our understanding of the vulnerability and adaptability of ecosystems to climate change in a regional scale. The result obtained in this study can provide a basis for environmental policy making.
